# Dual-modality Imaging of Angiogenesis in Unstable Atherosclerotic Plaques with VEGFR2-Targeted Upconversion Nanoprobes *in vivo*

**DOI:** 10.1007/s11307-022-01721-5

**Published:** 2022-05-23

**Authors:** Yan Fang, Ruichen Yang, Yi Hou, Yabin Wang, Ning Yang, Mengqi Xu, Sulei Li, Shan Gao, Min Jiang, Jingyang Fan, Yazhuo Hu, Zhenzhen Xu, Lei Gao, Feng Cao

**Affiliations:** 1 Medical School of Chinese PLA, Medical Department of Cardiovascular Disease in Sixth Medical Center & National Research Center for Geriatric Diseases, Chinese PLA General Hospital Chinese PLA General Hospital, Beijing, 100853 China; 2 Medical School of Chinese PLA, Department of Cardiology in Second Medical Center &, National Research Center for Geriatric Diseases, Chinese PLA General Hospital, Beijing, 100853 China; 3 Medical School of Chinese PLA, Medical Department of Cardiovascular Disease in Sixth Medical Center, Chinese PLA General Hospital, Beijing, 100853 China; 4 College of Life Science and Technology, Beijing University of Chemical Technology, Beijing, 100029 China; 5 Department of Cardiology, The Affiliated Hospital of Jiangnan University, Wuxi No.4 People Hospital, Jiangsu 21400 Wuxi, China; 6 Institute of Gerontology institute, Second Medical Center, Chinese PLA General Hospital, Beijing, 100853 China; 7 National Research Center for Geriatric Diseases, Chinese PLA General Hospital, Beijing, 100853 China

**Keywords:** Unstable atherosclerotic plaque, Angiogenesis, VEGFR2, Molecular imaging, Upconversion nanoprobes

## Abstract

**Aim:**

Angiogenesis plays a major role in atherosclerotic plaque development and instability. Our study aims to develop a novel optical and magnetic resonance (MR) dual-modality molecular imaging probe to early detect unstable plaques *in vivo* by targeting biomarkers of angiogenesis in murine models of atherosclerosis (AS).

**Methods:**

Immunofluorescence and western blot were used to detect the expression of Vascular Endothelial Growth Factor Receptor 2 (VEGFR2) in activated Human Umbilical Vein Endothelial Cells (HUVECs). After synthesis and identification of novel short peptide VRBP1-targeted VEGFR2, HUVECs were co-cultured with FITC-VRBP1 to test specific affinity of VRBP1. Then VRBP1-UCNPstargeting VEGFR2 were constructed by conjugating VRBP1 to the surface of NaGdF_4_:Yb,Er@NaGdF_4_ nanoparticles. The characterization of the nanoparticles was performed by transmission electron microscopy (TEM), distribution of size, hydrodynamic size, zeta potential, absorption spectra, emission spectra, imaging intensity of different concentrations, binding affinity and cytotoxicity of nanoprobes *in vitro*. The upconversion luminescence (UCL) and MR imaging were performed to identify unstable atherosclerotic plaque in ApoE^−/−^ mice *in vivo* and *ex vivo.* Morphological staining was used to verify AS model and angiogenesis, and Inductively Coupled Plasma-Atomic Emission Spectrometry (ICP-AES) was used to confirm accumulation of the nanoparticles after imaging.

**Results:**

After induced by hypoxia and ox-LDL, the expression of VEGFR2 in activated HUVECs was enhanced. FITC-VRBP1 can specifically bind to the HUVECs. Characterization of the nanoparticles showed that particles size is uniform with a stable structure, specific optical and MR signal, good binding affinity to VEGFR2 and low cytotoxicity. *In vivo* and *ex vivo* UCL imaging and quantitative analysis revealed that distinctive optical signal was observed in the regions of left carotid common arteries (LCCAs) of AS group after injection of VRBP1-UCNPs. Higher signal intensity on T1-weighted MR imaging appeared in the LCCA wall of AS group after injection. The results of morphological staining demonstrated angiogenesis in the atherosclerotic plaques, Gd ions in LCCAs, aortic arch and renal arteries bifurcations detected by ICP-AES confirmed accumulation of the nanoparticles in plaque.

**Conclusions:**

We successfully design and synthesize a novel UCNPs using peptide VRBP1 targeting to VEGFR2. *In vivo* imaging demonstrates that VRBP1-UCNPs can be used to perform optical/MR dual-modality imaging targeting angiogenesis in plaques, which is a promising technique to early detect unstable atherosclerosis.

## Introduction

Over the decades, considerable progress has been made in the prevention and treatment of cardiovascular diseases (CVD), but the mortality from CVD remains at high levels [1, 2]. The majority of acute cardiovascular events in patients is caused by occlusive thrombosis due to the rupture or erosion of atherosclerotic plaque [3, 4]. The formation of atherosclerotic plaque is a dynamic process, ranging from fatty streaks, atheroma and characteristic plaques. Accumulating evidence has proved the presence of angiogenesis in plaques may play a major role in atherosclerotic plaque growth and complications [5]. These neocapillaries are channels for inflammatory cells and lipid components to enter the plaques, which can accelerate the progression of the plaques. At the same time, the structure and function of new blood vessels is abnormal, which may induce intracerebral hemorrhage and increase the instability of plaques [6]. Thus, targeting plaque angiogenesis may be a useful strategy to early detect and evaluate unstable plaques.

Vascular endothelial growth factor (VEGF) family member receptors, belonged to the tyrosine kinase receptor family, include VEGFR-1, VEGFR-2 and VEGFR-3, as well as several other co-receptors. In the cardiovascular system, VEGFR-2 (also known as KDR, flk-1) is mainly expressed in endothelial cells, which is crucial to the maintenance of cardiovascular physiological function and the development of pathological processes of cardiovascular diseases. VEGFR-2 is the major positive-signal transducer for both physiological and pathological angiogenesis, highly expressed on the surface of endothelial cells of angiogenesis, but conserved in normal endothelial [7]. Thus, the high expression of VEGFR-2 is closely associated with instability of atherosclerotic plaque. For example, Margreet et al. confirmed that blocking VEGF-VEGFR2 signaling pathway can inhibit angiogenesis and increase plaque stability[8], and Wim Martinet et al. showed that inhibition of VEGF receptor signaling by axitinib attenuates intraplaque angiogenesis and plaque destabilization[9]. Therefore, VEGFR2 is a promising marker to evaluate angiogenesis in unstable atherosclerosis plaque. Numerous papers have reported imaging targeting VEGF receptors with VEGF-based tracers[10, 11], but a short peptide targeting to VEGFR2 is rarely used in imaging. Using high-throughput bacterial display methods, a peptide named VRBP1 was identified as an effective molecule targeting to VEGFR2 for diagnosis and therapy[12].

Molecular imaging technology can non-invasively monitor the dynamic changes of the diseases with the molecular markers, which makes it possible to *in vivo* observe the pathologic progress of the disease. Moreover, it can achieve early accurate diagnosis and targeted therapy of diseases by intervening the specific molecule target [13]. To implement above aims, many efforts have been made to improve molecular probe materials. Recent researches showed that novel upconversion luminescent (UCL) material doped with rare earth elements such as ytterbium (Yb) and erbium (Er) has many advantages, such as high chemical stability, low toxicity, deep tissue penetration, no damage to biological tissue, nearly zero background fluorescence interference and high imaging sensitivity [14]. Due to the unique optical and intrinsic paramagnetic properties, UCL material can be an ideal candidate for constructing molecular imaging nanoprobes [15].

Our study attempted to use VEGFR-2 as a molecular target of angiogenesis in atherosclerotic plaque and design a novel molecular imaging probe via conjugating VEGFR-2-targeted short-peptide VRBP1 with upconversion nanoparticles (UCNPs), to realize dual-modality optical and MR imaging in murine atherosclerotic plaque *in vivo*.

## Materials and Methods

### Animals and procedures

Six-week-old male mice were purchased and acclimatized for one week (Vital River Laboratory Animal Technology Co., Beijing). The mice were allocated into 2 groups with 24 in each. (1) Control group (Con): C57BL/6 genetic mice were fed with standard laboratory chow diet for 16 weeks. (2) Atherosclerosis group (AS): apolipoprotein E-deficient (ApoE^−/−^) mice (C57BL/6 J genetic background) were performed a partial ligation at their left carotid common arteries (LCCAs), followed by feeding with a high-fat, high-cholesterol diet (standard laboratory chow 83.55% + lard 15% + cholesterol 1.25% + sodium cholate 0.2%, Vital River, Beijing) for 16 weeks. The animal experiment protocol was approved by the Experimental Animal Welfare Ethics Committee of PLA General Hospital.

### Expression of VEGFR2 in HUVECs induced by hypoxia and ox-LDL

HUVECs (CRL-1730, ATCC, America) were cultured in Hyclone DMEM (Wolcavi, Beijing) with high glucose supplemented with 10% fetal bovine serum (Gibco, Grand Island, America), streptomycin and penicillin (Sigma, America), and maintained in humidified environment containing 5% CO_2_ and air at 37 °C. After stimulated, respectively, by hypoxia, ox-LDL (10 μg/mL, Solarbio, Beijing), and double stimulation of ox-LDL and hypoxia for 48 h, the expression level of VEGFR2 in activated HUVECs was detected by immunofluorescence and western blot.

### Synthesis and identification of the VEGFR2-targeted short-peptide VRBP1

The VEGFR2-targeted peptide VRBP1 was synthesized by the solid-phase peptide synthesis method. The amino acid sequence of VRBP1 was as follows: Tyr-Asp-Gly-Asn-Ser-Phe-Tyr-Glu-Met-Trp-Gly-Val-Lys-Pro-Ala-Ser-Glu-Ser. For verifying the targeting property of VRBP1, the synthesized VRBP1 and control non-targeted peptide IgG were coupled with FITC (fluorescein isothiocyanate isomer, Sigma, American), respectively. After induced by hypoxia and ox-LDL (10 μg/mL, Solarbio, Beijing) for 48 h, HUVECs were incubated with VRBP1-FITC and IgG-FITC for another 4 h. Then DAPI was used for staining nuclei in the HUVECs. The fluorescence was imaged by a confocal fluorescence microscope (Olympus, Japan), and image data were analyzed by ImageJ software.

### Design and Synthesis of VRBP1-UCNPs

The PEGylated NaGdF_4_:Yb,Er@NaGdF_4_ UCNPs with surface maleimide groups were prepared according to the method previously reported[15]. Subsequently, the VEGFR2-targeted probes were prepared by mixing the thiol group (-SH) terminated VRBP1 with PEGylated NaGdF_4_:Yb,Er@NaGdF_4_. The resultant VRBP1-UCNPs were transferred into 1 × PBS buffer stored at 4 °C in the dark for further use.

### Characterizations of nanoparticles

TEM (transmission electron microscopy, JEM-2100, JEOL, Tokyo, Japan) images were captured to determine the size and morphology of the NaGdF_4_:Yb,Er and NaGdF_4_:Yb,Er@NaGdF_4_ nanocrystals, and their size distributions. The emission spectra of nanoparticles were recorded in a Cary Eclipse fluorescence spectrophotometer equipped with a 980-nm CW laser diode (2 W) as the excitation light source. Dynamic light scattering (DLS, Zetasizer Nano ZS90, Malvern Instruments JEM-100CXII, Worcestershire, UK) was used to measure the hydrodynamic size and zeta potential of VRBP1-UCNPs. Optical and MR signal intensity of nanoparticles (VRBP1-UCNPs, abbreviated as VNPs; UCNPs, abbreviated as NPs) at different concentrations was detected and quantitatively analyzed by a modified upconversion luminescence imaging system IVIS Spectrum (PerkinElmer, America) and a 7.0 T animal MRI system (Bruker Biospec 70/20 USR, Germany), respectively.

### *In vitro* binding and cytotoxicity of VRBP1-UCNPs

After stimulated by hypoxia and ox-LDL (10 μg/mL) for 24 h, the induced HUVECs were incubated with VRBP1-UCNPs and non-targeting IgG-UCNPs for another 12 h, respectively. Subsequently, the cells were rinsed with PBS and fixed with 4% formaldehyde. After that, the cells were stained with the azide chromogenic agent for 0.5 h to identify binding of VRBP1-UCNPs to induced HUVECs. The imaging of cells was carried on a light microscope (Olympus, Japan).

HUVECs were incubated with VRBP1-UCNPs of concentration 0, 5, 10, 15, 20, 25, and 30 ug/mL for 24 h, and CCK-8 (Cell Counting Kit-8, Solarbio, Beijing) was used to evaluate the cytotoxicity of VRBP1-UCNPs.

### *In vivo *and *ex vivo* UCL imaging and MR imaging

Mice were anaesthetized by isoflurane, and then *in vivo* UCL imaging was conducted before and at 30 min, 60 min, 120 min after intravenous injection of 100 μL VRBP1-UCNPs solution (2.5 mg/mL) through tail vein. At 120 min, the major vessels were isolated from mice to perform *ex vivo* UCL imaging. Images were captured with a modified animal UCL imaging system IVIS Spectrum (PerkinElmer, American) with the imaging parameters set as follows: binning, 2; F/stop, 2; exposure time, 18.0 s; excitation laser, 980 nm; emission filter, 660 nm. The UCL signals were quantitatively analyzed by Living Image 4.0 software (Caliper, MA, America).

*In vivo* T1-weighted MR imaging was conducted before and 120 min after intravenous injection of 100 μL VRBP1-UCNPs solution (2.5 mg/mL). MR images of the atherosclerotic plaques were acquired on a 7.0 T animal MRI system (Bruker BioSpin MRI GmbH, Germany) with parameters set as follows: vertical field of view, 30 mm; horizon field of view, 30 mm; base resolution, 256 × 256; slice thickness, 0.5 mm; time to echo, 9 ms; repetition time, 1000 ms. The imaging data were analyzed by VivoQuant 2.0 software, and the representative MR images of plaque were located in the LCCA walls.

### Histological validation and *ex vivo* analysis

After UCL and MR imaging, the major arteries gross specimens of the mice were separated, including left and right common carotid artery, left subclavian artery, aortae, and iliac artery. Fixed arteries tissue with 4% formaldehyde for 20 min and then stained with oil red O dye for lipid plaques. To validate unstable atherosclerotic plaque, the LCCAs were harvested from mice for H&E staining, collagenous fiber Sirius red, and connective tissue Masson-trichrome staining, respectively.

Immunohistochemistry staining in section of carotid artery was conducted to verify the expression of angiogenesis marker α_ν_β_3_ in the atherosclerotic plaques. Microscopic images were taken with an inverted microscope (Olympus, Japan). The expression of α_ν_β_3_ in plaques was quantitatively analyzed by ImageJ.

Laser confocal microscopy with a 980-nm laser diode as the excitation light source was used to detect VRBP1-UCNPs signals in atherosclerotic plaque. Besides, LCCAs, aortic arches, and renal arteries bifurcations were harvested and digested by mixed acid. Then Gd concentration in LCCAs, aortic arches, and renal arteries was determined quantitatively by Inductively Coupled Plasma-Atomic Emission Spectrometry (ICP-AES).

### Statistical Analysis

Continuous variables following a normal distribution were expressed by mean ± standard deviation (M ± SD). Proportions variables were assessed with a Chi-square test. Multiple group comparisons were made by one-way analysis of variance (ANOVA). Two independent groups comparisons were analyzed using the Student’s t test. Two-sided tests were used throughout the experiment. P < 0.05 was considered statistically significant. GraphPad Prism-6 software was used for all data analysis.

## Results

### Expression of VEGFR2 in activated HUVECs and identification of peptides VRBP1 targeting to VEGFR2

**Fig. 1:**
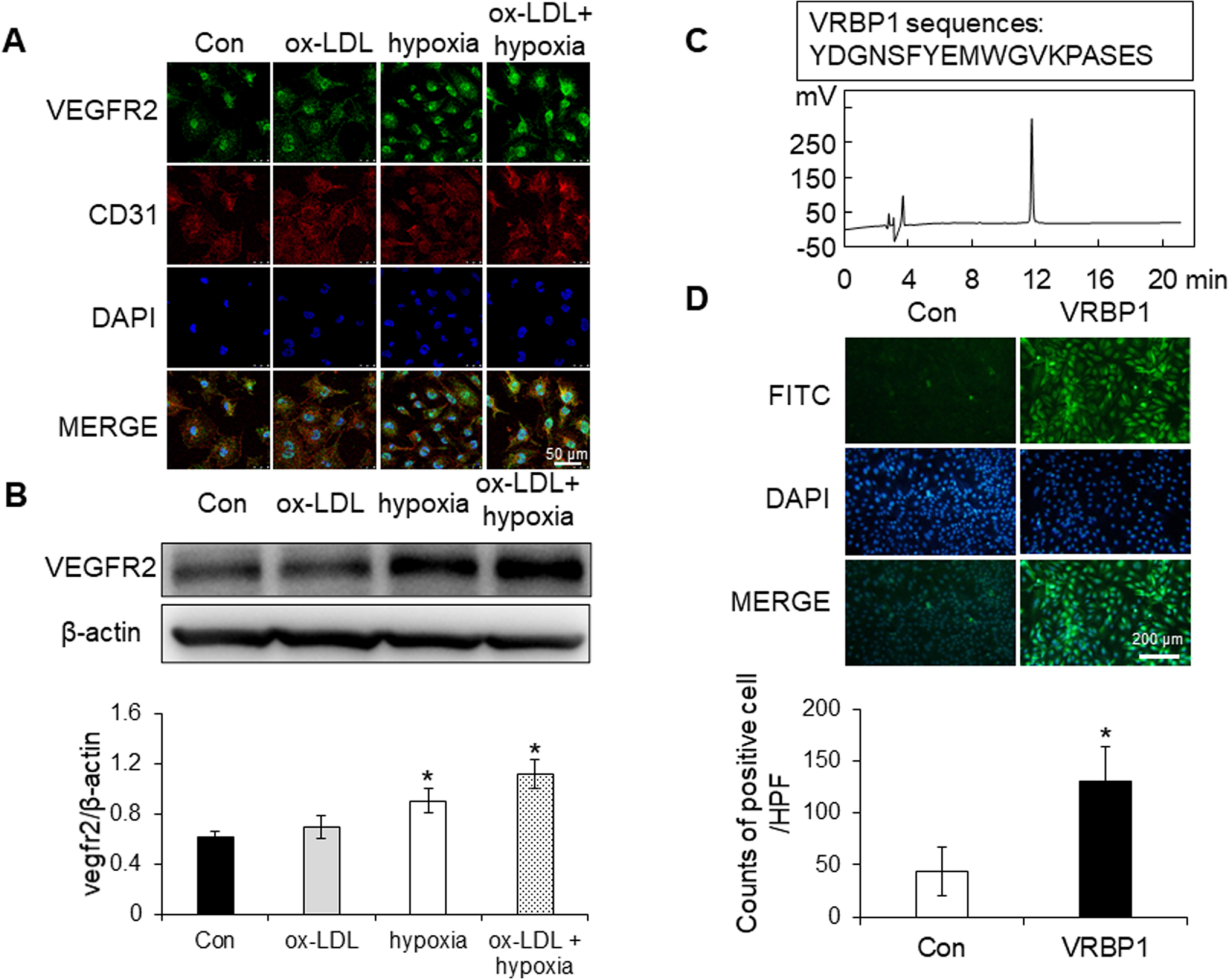
VEGFR2 expression in activated HUVECs and identification of peptides VRBP1 targeting VEGFR2. **A** VEGFR2 expression in HUVECs detected by cell immunofluorescence. (Scale bar = 50 μm). **B** VEGFR2 expression in HUVECs detected by western blot. **C** Identification purity of synthetic peptides VRBP1 targeting VEGFR2 by HPLC. **D** Targeting ability of peptides VRBP1 detected by cell direct immunofluorescence (scale bar = 200 μm) and relative quantitative analysis *in vitro*. (Values are expressed as the mean ± SE, n = 3 per group, *P < 0.05 vs. the control.)

As shown in Fig. 1A, the results of cell immunofluorescence indicated that the expression of VEGFR2 in HUVECs stimulated by hypoxia and ox-LDL was higher compared to control group. Furthermore, western blot analysis showed that the VEGFR2 expression in HUVECs stimulated by hypoxia and ox-LDL was more than control group (*p < 0.05). The HPLC revealed a peak value of 11.788 min, which indicated purity of short-peptide VRBP1 more than 95.88% in (Fig. 1C). According to the immunofluorescence assay provided in Fig. 1D, VRBP1-FITC bound more on HUVECs stimulated by hypoxia and ox-LDL than IgG-FITC (*p < 0.05), and VRBP1 can be used as a targeting peptide with a good selective affinity targeting to VEGFR2 receptor.

### Synthesize and characterization of the VRBP1-UCNPs

**Fig. 2:**
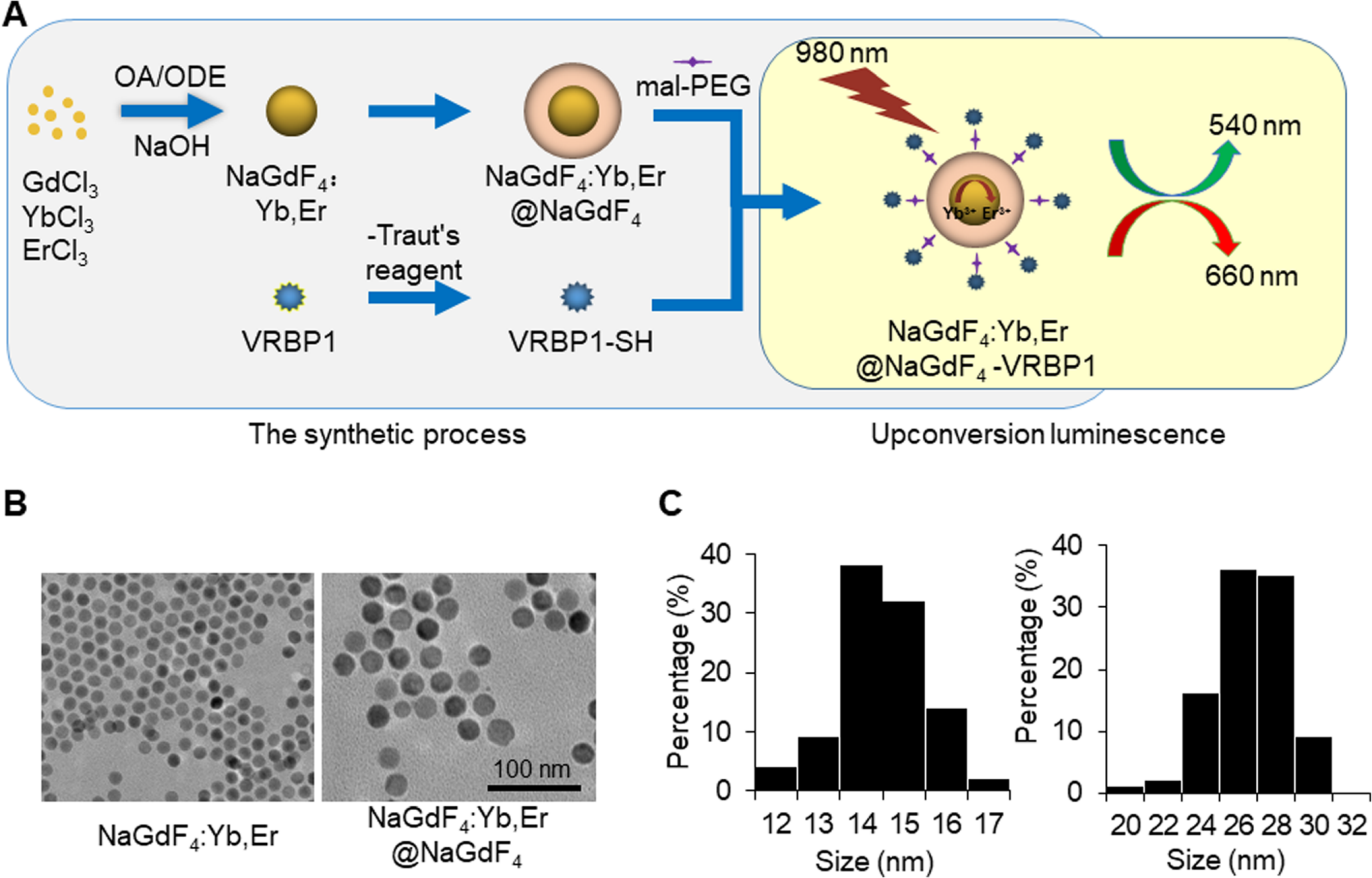
Synthesize of the VRBP1-UCNPs, morphology, and size characterization of the nanoprobes. **A** Scheme of the VRBP1-UCNPs synthetic process. **B** The morphology and size of UCNPs were characterized by transmission electron microscopy (TEM) (scale bar = 100 μm). (**C**) The distributions of particle size of UCNPs

The schematic VRBP1-UCNPs synthesis process is illustrated in Fig. 2A. NaGdF_4_ nanoparticles doped with Yb^3+^ and Er^3+^ exhibit upconversion luminescence at 540 nm (green) and 660 nm (red) under 980-nm laser excitation, and the core@shell structure (NaGdF_4_:Yb,Er@NaGdF_4_) can improve the UCL efficiency. Gd-based VRBP1-UCNPs exhibit paramagnetic property similar to Gd-DTPA, a contrast agent widely used in clinical MRI practice; therefore, the nanoprobe can be used to enhance the MR signal contrast. Conjugated targeted VRBP1 peptide enables the probe to specifically bind with VEGFR2 molecules.

The TEM image and distribution histogram of NaGdF_4_:Yb,Er and NaGdF_4_:Yb,Er@NaGdF_4_ nanocrystals in Fig. 2B and 2C showed that both nanoparticles were almost monodispersed with an average size of 15.0 ± 1.0 nm and 25.7 ± 1.9 nm, respectively.

**Fig. 3:**
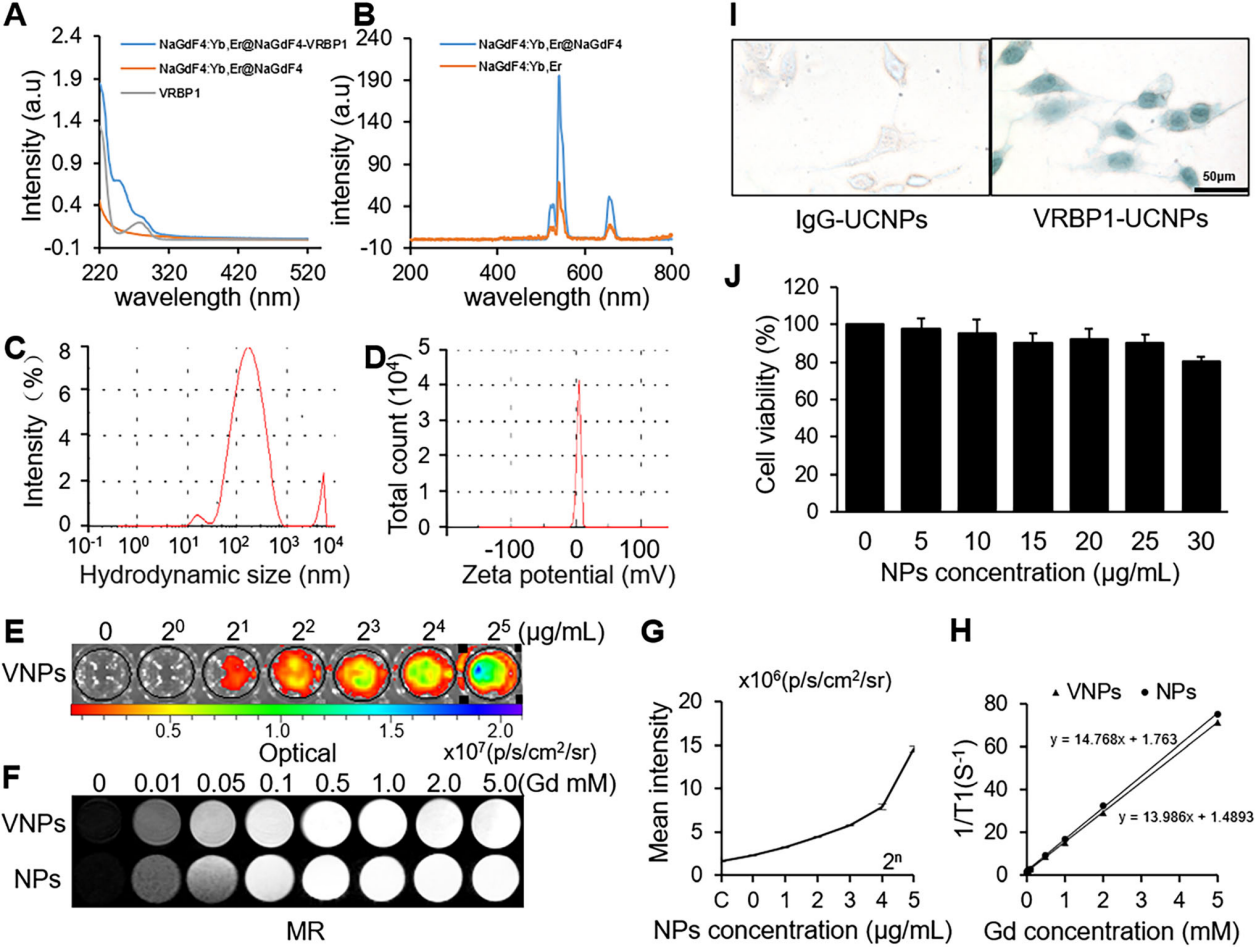
Characterization, cytotoxicity, binding ability of the nanoprobes. **A, B** The absorption spectra of VRBP1 and UCNPs, and emission spectra of the UCNPs under 980-nm laser excitation. **C, D** Hydrodynamic size and zeta potential of VRBP1UCNPs. **E** Upconversion luminescent (UCL) and **F** magnetic resonance (MR) imaging and **G, H** quantitative analysis at different concentrations of nanoparticles. **I** Binding ability to HUVECs of VRBP1-UCNPs identified by the azide chromogenic agent dyeing (scale bar = 100 μm). (**J**) Cytotoxicity of VRBP1-UCNPs evaluated by CCK-8 assay (values are expressed as the mean ± SE, n = 6 per group)

The absorption spectra of the VRBP1, the NaGdF_4_:Yb,Er@NaGdF_4_, and the NaGdF_4_:Yb,Er@NaGdF_4_-VRBP1 probe are provided in Fig. 3A. It is clear that the absorption spectra of NaGdF_4_:Yb,Er@NaGdF_4_-VRBP1 contained the specific absorption spectra of NaGdF_4_:Yb,Er@NaGdF_4_ and VRBP1, indicating a successful conjugation of NaGdF_4_:Yb,Er@NaGdF_4_ with VRBP1. The emission spectra of UCNPs under 980-nm laser are shown in Fig. 3B. Two main emission peaks were recorded at 540 nm and 660 nm, corresponding to the green and red spectral region. The luminescence intensity of core@shell structural NaGdF_4_:Yb,Er@NaGdF_4_ was much higher than that of the core NaGdF_4_:Yb,Er nanoparticles.

After conjugated with VRBP1, the nanoprobe can disperse in the aqueous medium with stable physicochemical properties and no apparent agglomeration. The Z-average value of hydrodynamic size of VRBP1-UCNPs is 157.3 nm, max peak in the intensity particle size distribution plot is 190.6 nm, and particle size dispersion index is 0.428, as shown in Fig. 3C. Zeta potential is 0.34 mV (Fig. 3D), which is approximately electrically neutral; thus, the probes are believed to diffuse across vascular basement membrane into the plaque.

UCL and MR imaging *in vitro* upon concentrations of nanoparticles in Fig. 3E and 3F showed that the signal intensity increased with the concentration. As shown in Fig. 3H, the relaxivity (r1) of VRBP1-UCNPs was calculated to be 13.986 mM^−1^ s^−1^, slightly lower than that of UCNPs (14.768 mM^−1^ s^−1^). The MRI results showed that the T1 relaxation time was significantly affected by nanoparticles, and there was a strong linear correlation between the concentration and the signal intensity.

### *In vitro* binding and cytotoxicity of VRBP1-UCNPs

The binding of VRBP1-UCNPs to HUVECs induced by Ox-LDL and hypoxia is shown in Fig. 3I. The uptake amounts of VRBP1-UCNPs were significantly more than that of non-targeted IgG-UCNPs in HUVECs, indicating the specific binding of VRBP1-UCNPs *in vitro*.

The results of CCK-8 assay are provided in Fig. 3J. Compared with control group, the survival rate of cell incubated with VRBP1-UCNPs showed no significant variation (*p < 0.05) as the concentration of nanoparticles increases from 5 μg/mL to 30 μg/mL, indicating a low cytotoxicity of the probes.

### *In vivo *and *ex vivo* UCL and MR imaging of atherosclerotic plaque

**Fig. 4:**
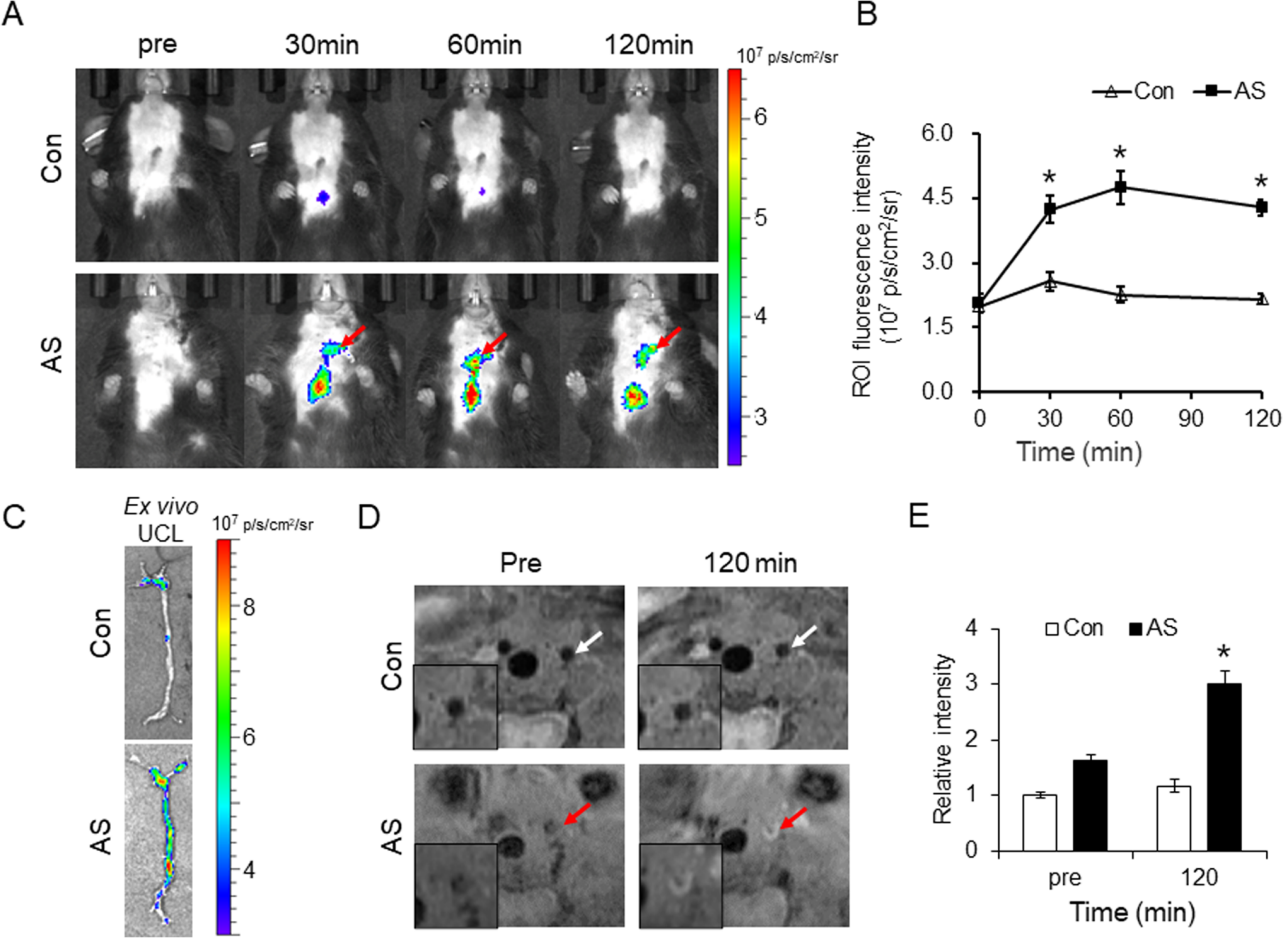
*In vivo* and *ex vivo* UCL and MR imaging. **A** *In vivo* UCL imaging at different time points after injection of the VRBP1-UCNPs by IVIS spectrum. **B** Quantitative analysis of UCL images in regions of LCCAs. **C** *Ex vivo* UCL imaging of isolated blood vessels. **D** *In vivo* T1-weighted MR imaging before and 120 min after injection of the VRBP1-UCNPs by 7.0 T animal MRI system. **F** Relative quantitative analysis of MR images in regions of LCCAs. (Con, control; AS, atherosclerosis; values are expressed as the mean ± SE, n = 6 per group, *P < 0.05 *vs*. the pre-group)

As shown in Fig. 4A, *in vivo* images of UCL at 660-nm emission were acquired after intravenous injection of VRBP1-UCNPs under 980-nm laser excitation. As we all known, atherosclerosis is prone to occur at the bifurcation of blood vessels in ApoE^−/−^ mouse (AS group), such as aortic arch, abdominal aortic bifurcation, and renal arteries bifurcation. Because the partial ligation was performed at left common carotid arteries (LCCAs) in AS group to alter hydrodynamic shear stress, atherosclerosis is also prone to occur there. Therefore, obvious UCL signals were observed at location of LCCAs (pointed by red arrows) and aortic arch in AS group. For hair and thick skin covering abdomen, it is difficult to observe UCL signals in abdominal aorta and renal artery bifurcation. In control group, C57BL/6 mice can only develop lipid streaks, not atherosclerotic plaques in artery walls; hence, VRBP1-UCNPs barely accumulated in the walls of LCCAs, which cannot produce strong enough signal to be observed under the current scale bar in control group. Because the atherosclerosis lesion in aortic arch is generally more severe than LCCA, by self-comparison, a little signal in the location of aortic arch was observed despite no signal in LCCA. Overall, the results revealed that the signals located at LCCAs were stronger in AS group than that in control group. Quantitative analysis (Fig. 4B) further showed that the signal intensity was significantly higher after injection of VRBP1-UCNPs than that before injection in AS group (*p < 0.05), which demonstrated pretty good UCL imaging capability of UCNPs to specifically identify plaques.

*Ex vivo* UCL imaging of blood vessels in Fig. 4C showed that amounts of UCL signals were enriched in the aortic arch and LCCAs, which was consistent with the signals *in vivo* imaging. And the signals located at abdominal aortic and renal arteries bifurcation covered by hair and thick skin when *in vivo* imaging, can be observed when *ex vivo* imaging.

As shown in Fig. 4D, thickened artery walls and atherosclerotic plaques of the LCCA were pointed by red arrows in AS group, while intact artery walls without plaques of the LCCAs were pointed by white arrows in control group, in T1-weighted MR imaging *in vivo*. The narrowed lumen of LCCAs in AS group was consistent with previous operation of partial ligation in LCCAs altering blood flow shear stress to cause atherosclerosis. In AS group, the outlines of atherosclerotic plaques in LCCA walls were curved with a significantly narrowed lumen, in which the enhanced T1-weighted MR signal was obvious at 120 min after injection of VRBP1-UCNPs, compared with that before injection, showed in enlarged images in bottom left. In control groups, for intact vessel walls without atherosclerotic plaque in LCCAs, no enough VRBP1-UCNPs were accumulated there, and no obvious signal changes were observed after injection of VRBP1-UCNPs, compared with that before injection. Furthermore, as shown in Fig. 4E, the quantitative analysis results of T1-weighted MR images indicated that the relative signal intensity of the LCCAs walls at 120 min was significantly higher than that before injection of VRBP1-UCNPs in AS group (*p < 0.05).

### Histological validation and *ex vivo* analysis

**Fig. 5:**
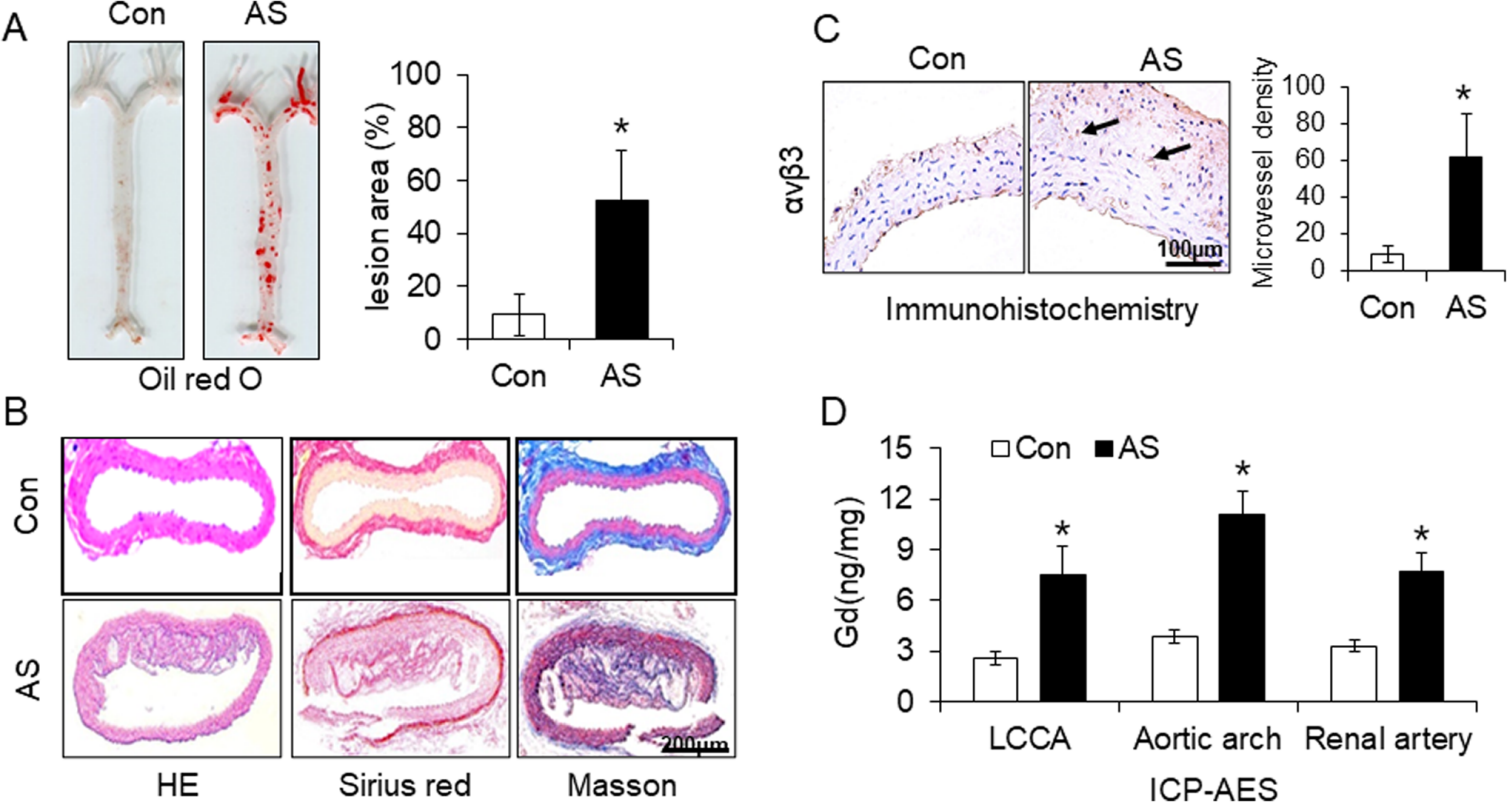
Histological validation and** ex vivo** analysis confirmed angiogenesis and nanoprobes in atherosclerotic plaque. **A** Oil red O and **B** HE, sirus red, and masson staining showed atherosclerotic lesions in arteries of ApoE^−/−^ mice (scale bar = 50 μm). **C** Angiogenesis confirmed by α_ν_β_3_ immunohistochemistry staining and relative quantitative analysis (scale bar = 100 μm). **D** Detection of Gd in LCCAs, aortic arch, and renal arteries bifurcations by ICP-AES and quantitative analysis. (Con, control; AS, atherosclerosis; values are expressed as the mean ± SE, n = 6 per group, *P < 0.05 *vs*. the control)

Oil red O staining in Fig. 5A indicated abundant lipid-rich plaque in the major arteries isolated from AS mice, especially in LCCA in AS group. H&E, Sirius red, and Masson-trichrome staining of the carotid arteries from above tissue in Fig. 5B revealed typical features of unstable atherosclerotic plaque morphology, including thickened intima, lager lipid-rich plaque protruding into lumen, thin fibrous cap, cholesterol crystals, and inflammatory infiltration, which validated a successful unstable atherosclerotic plaque model in mice (Fig. 5B).[5] For further verifying angiogenesis in plaque, the immunohistochemistry results of ανβ3 and quantitative analysis in Fig. 5C showed more angiogenesis in the plaques of the AS group compared with control group (*p < 0.05).

Due to the limited number of probes on a single slice (6 μm) and the lack of amplification of fluorescence, the signal is not strong enough to be observed by confocal microscopy after irradiation with 980-nm excitation light. Then another method, ICP-AES, with higher sensitivity at the nanogram level, was used to quantify Gd element in the LCCAs, to indirectly verify that the probes are enriched in plaques. As shown in Fig. 5D, Gd concentrations in LCCAs, aortic arch, and renal arteries bifurcation of AS group are significantly higher than that in control group (*p < 0.05), indicating nanoparticles accumulation in plaques in AS group.

## Discussion

In the current study, we designed and synthesized a novel dual-modality imaging probe based on NaGdF_4_:Yb,Er@NaGdF_4_ nanoparticles using both MR and UCL imaging for *in vivo* visualization of atherosclerotic plaques. Via the specific interaction of VRBP1 with VEGFR2, angiogenesis in unstable plaques in murine model of atherosclerosis was successfully detected. Histological analysis and ICP-AES detection further validated the excellent specificity of the nanoprobe for atherosclerosis diagnosis.

As known, several invasive molecular imaging techniques have been used to evaluate the progress of atherosclerosis, such as PET, CT, MR, ultrasound, and optical imaging. Due to high sensitivity, PET or PET/CT imaging radiotracer including ^68^ Ga-DOTATATE, ^[18F]^FDG, ^[18F]^NaF, was used as novel markers for inflammation or calcifications in atherosclerotic plaques [16, 17]. But radiation exposure limits the widespread use of PET imaging. Some studies demonstrated that contrast-enhanced ultrasound can be used to detect intraplaque neovascularization with both high sensitivity specificity [18]. However, several drawbacks exist when applying ultrasound, such as spatial resolution, motion artifact, and limited tissue penetration. [19] MR has been considered as a useful imaging tool in analysis of experimental atherosclerotic plaque, due to its excellent spatial resolution, soft tissue contrast, and 3-dimensional imaging capabilities. But the relatively lower contrast sensitivity left lots of limitations of MRI to detect plaques. Even optical imaging with high sensitivity was limited by distance-dependent manner, resulting in semiquantitative near-infrared (NIR) fluorescent signal assessment [20]. Therefore atheroma larger than a typical coronary artery did not produce detectable NIRF signal *in vivo*. Therefore, the combination of MRI and optical imaging strengths is an appropriate approach for non-invasively monitoring the dynamic changes of plaques. Furthermore, Er^3+^-doped upconversion materials sensitized by Yb^3+^ showed more useful features including long luminescence lifetime (μs–ms range), narrow absorption and emission band widths (< 10 nm), high quantum yields, low toxicity, and the deep tissue penetration with reduced photo-damage and auto-fluorescence for the NIR exciting lights within tissue optical windows [21]. Hence, Gd-based UCNPs with high performance of MRI and UCL imaging were chosen as ideal probes for dual-modality optical/MRI imaging.

To verify the probe properties, a series of characterizations of UCNPs was conducted, especially chemical stability, cytotoxicity, biocompatibility, and active targeting ability. Various of TEM images and size distributions revealed UCNPs properties of small hydrated size, good stability, and almost no particles aggregation, similar to our previous report [22], which make particles easily penetrate into plaques by injured endothelium or vasa vasorum. The emission and absorption spectra confirmed the properties of narrow absorption and emission band widths (< 10 nm), which were also evidenced by our previous study. CCK-8 assay suggested that UCNPs are non-toxic and of good biocompatibility to the endothelial cells below a certain threshold, suitable to be an MRI contrast agent for atherosclerotic plaques imaging *in vivo*. After conjugated with VEGFR2-targeted peptide VRBP1, imaging *in vitro* and *in vivo* showed efficiently active targeting ability of UCNPs.

Angiogenesis is a dynamic process regulated by a delicate balance between angiogenic and angiostatic factors, which finally lead to changes in vascular networks [23]. Adequate evidence demonstrated that the neoangiogenesis presented in atherosclerotic plaques and played a critical role in the progression of atherosclerotic plaque [6, 24]. There exited a strong association between the density of vasa vasorum in adventitial vessel walls and severity of plaque formation, and vasa vasorum is an early event in initiation of atherosclerosis [25]. Furthermore, adventitial administration of VEGF elicits neoangiogenesis and plaque formation, whereas inhibiting angiogenesis by TNP470 attenuated plaque growth [6]. Clinical research confirmed that intraplaque angiogenesis with hemorrhages was mainly associated with thin-cap fibroatheroma (TCFA), macrophage infiltration, and large necrotic cores, which are characterizations of unstable plaques [26]. For the peculiar role of angiogenesis in unstable plaques, it is worthy of discovery of novel biomarkers for angiogenesis imaging to provide new insights in detection and diagnosis of atherosclerosis in early stage.

Researches showed that a large number of factors participate in the regulation of angiogenesis in atherosclerotic plaques, and VEGF is a key regulator in endothelial cells. VEGF binding to VEGFR2 initiates autophosphorylation of VEGFR2, increasing VEGFR2 tyrosine kinase activity, triggering the recruitment of several signaling molecules, inducing the activation of downstream signaling enzymes including ERK1/2, Akt, and eNOS, and leads to proliferation, migration, and tube formation of endothelial cells, finally stimulating angiogenesis [27, 28]. Moreover, intervening VEGFR2 signaling pathway by VEGFR2 vaccination has been demonstrated to block angiogenic responses and attenuate the progression of advanced atherosclerotic lesions, which is a target of antiangiogenic therapy [29]. Based on the above analysis, we chose VEGFR2 as a biomarker for molecular imaging of angiogenesis in atherosclerotic plaques in our study.

Firstly, for targeting VEGFR2, a novel short peptide VRBP1 was constructed, which can specifically bind to induced HUVECs with high express of VEGFR2. And then, for dual modality, we synthesized VRBP1-UCNPs by conjugating VRBP1 to PEG-coated UCNPs and validated good binding ability of VRBP1-UCNPs to HUVECs *in vitro* and further implemented dual-modality UCL/MRI imaging of unstable atherosclerotic plaques *in vivo,* which was confirmed by histological validation and *ex vivo* analysis. So, we successfully achieved visualization of angiogenesis in unstable plaques in murine model of atherosclerosis with VEGFR2-targeted upconversion nanoprobes *in vivo*, which is promising in detection and diagnosis of atherosclerosis in early stage.

There are some limitations of this probe. The applications of UCNPs are limited to laboratory use, biocompatibility, and reduced toxicity that remain to be improved.

## Conclusion

We have synthesized a VEGFR2-targeted peptide VRBP1 and developed a novel dual-modality probe based on VRBP1 and UCL materials to perform optical/MR imaging in murine models, which allowed non-invasive assessment of angiogenesis in plaques and early diagnosis of unstable atherosclerosis.
